# The Ethics of Aesthetic Surgery

**DOI:** 10.4103/0974-2077.63396

**Published:** 2010

**Authors:** SR Mousavi

**Affiliations:** *Professor of Surgery and Cosmetic and Ethics, Shohada Medical Center, Tajrish University of Shahid Beheshti Medical Sciences, Tehran Iran*

**Keywords:** Autonomy, cosmetic surgery, ethics

## Abstract

Advances in plastic and reconstructive surgery have revolutionized the management of patients suffering from disfiguring congenital abnormalities, burns and skin cancers. The demand for aesthetic surgery has increased in recent years, as our culture has become more concerned with image and appearance. Several ethical considerations such as patient's right for informed counseling, beneficience and maleficience need to be given careful consideration.

## INTRODUCTION

In the independent healthcare sector, aesthetic surgery has increased in popularity, reflecting increased consumer demand. Many regard aesthetic surgery as a panacea for their personal and relationship difficulties. Active and aggressive media, which were almost absent 50 years ago, have made our society ambitious and globalized the perception of what is attractive, desirable, and sexy. In addition, our lifestyle has changed with the fast growing offer of leisure activities. This may reflect more popular and modern image obsessed culture, but it can also be due to deep-rooted insecurities.

In the past few years the abuse of ethical principles in plastic surgery has become increasingly noticeable.[1] In 1979, Beauchamp and Childress published *Principles of Biomedical Ethics*, in which they presented four ‘principles’ that have since been adopted as the ethical basis for contemporary medical practice. They argued that these principles “bridged” high-level moral theory and what they described as “low-level common morality”. These principles included: respect for autonomy, beneficience, non-maleficience and justice. While these principles were developed to guide physicians treating those suffering from ill health, they provided the ethical framework which underpinned modern surgical practice.[2] As the concepts of beauty undergo changes, wrinkles, fat deposits and sun-damaged skin no longer fit into our concept of a neat society.[3] As these real or perceived ageing problems received greater attention from patients and doctors, the ethical considerations also need to be focused upon. Enhancement versus therapy, risks, patient autonomy, beneficence and informed consent are issues that need to be reconsidered and emphasized, when considering aesthetic surgery.[4] This article outlines these ethical concepts

## RESPECT FOR PATIENT AUTONOMY

In general, competent adults have the right to decide whether they wish to undergo a surgical procedure or not. The patients' wishes and thereby their right to an informed decision must be respected, provided they have been given sufficient information. Information must include the risks of surgery together with alternative options. These principles apply even more to aesthetic surgery where patients are not suffering from any ‘illness’. Elective aesthetic treatments, which may lead to long-term adverse effects on body function and health, involve serious ethical concerns. In such situations, the patient's right for autonomy may contradict the physician's principle of non-maleficence (see later in text), and therefore, proper consideration is needed before deciding on such treatments.[5] Surgeons must therefore ensure that the patients' expectations are realistic. Surgeons need to explain the probable benefits of surgery, alternative non-surgical options, as well as, the risks of surgery and anaesthesia.

## BENEFICIENCE

This principle requires that medical practitioners act in the patients ‘best interests’. Undertaking surgery to improve a patient's self-image and esteem is acceptable. However, defining the patient's best interests can be very difficult. Many people experience real pain, discomfort, social handicap and suffering because they are self-conscious about their appearance. These groups may benefit from aesthetic surgery. Body Dysmorphic Disorder (BDD) is a psychiatric syndrome, characterized by a pre-occupation with a non-existent or minimal cosmetic ‘defect’ associated with persistent attempts to have the defect surgically corrected. BDD is increasingly recognized, and may be becoming more prevalent.

## NON-MALEFICIENCE

This principle ensures that a aesthetic surgeon never acts against the patients' best interests or in a way that may harm a patient. Consultant aesthetic surgeons may decline to operate on patients if they do not believe that the surgery is in the patients' best interests. Aesthetic surgeons should be reluctant to operate on those with unrealistic expectations, as the risks of surgery may outweigh any benefits. Patients with serious health problems are at increased risk of suffering complications under general anaesthesia, and again the risks may outweigh the benefits. All such assessments need to be made on an individual basis. In the past, there has been a perception that surgeons have a potential conflict of interest in the independent sector. No surgeon should ever proceed with an operation merely for personal pecuniary gain. All aesthetic surgeons need to take their duty of care to their patients very seriously.

Surgery should only be undertaken in premises that are fully-equipped, with resuscitation facilities and staff trained in advanced life support. Surgeons, clinics and hospitals providing aesthetic surgery should be registered with the appropriate regulatory authorities.

## JUSTICE

This principle requires doctors to ensure that medical care is available to all. Equitable access to healthcare is regarded as a basic human right. However, resources are limited and it is not possible for any health service to provide aesthetic surgery for all those who would like it. ‘Rationing’ takes place on the basis of clinical need. Inevitably, this introduces subjective judgements about whose need is greater. In the private sector, those who can afford to pay, undergo surgery.

## SOCIAL CONSIDERATIONS

Body image, in our consumer societies, has become much more important, and its new modelizations magnified by media, affect the plastic surgeon with ethical questions. One of the ethical dilemmas in aesthetic surgery is the open advertising of the surgeon. On the other hand, the very nature of the individual who specializes in this kind of surgery may be the principal basis for publicity. Such abuse of professional ethics is usually explained by claiming that the public should be properly informed.[1]

Requests for aesthetic surgery occur in all social classes.[6] However, as stated earlier, because of economic considerations, there is no social system in the world that covers aesthetic surgery, except for some definite interventions.

## THE ADOLESCENT PATIENT

The deformity, physical and emotional maturity and the desired outcome for each adolescent patient must be carefully evaluated before any decisions are made. Additional consultations and long discussions before Aesthetic surgery are always necessary. Sometimes, however, the real question is: Is it ethical *not* to operate on an adolescent patient?[7]

With appropriate patient selection, aesthetic surgery can offer excellent cosmetic results. Patients must undergo thorough pre-operative assessment and counseling before surgery. This may require expert psychological assessment.

## CONCLUSION

At present, aesthetic surgery is passing through an identity crisis as well as an acute ethical dilemma. A closer look from an ethical viewpoint makes it evident that the doctor who offers aesthetic interventions faces many serious ethical problems, which have to do with the identity of the surgeon as a healer.[8] Aesthetic surgery makes profit from the ideology of a society that serves only vanity, youthfulness and personal success, and one which is losing sight of the real values. The real value of a person cannot be reduced to his / her appearance, and medicine as an art, should feel the obligation to resist these modern ideologies and should attempt to help people get a more authentic attitude about themselves.[9] Plastic surgeons must resist aesthetic measures in children and adolescents, particularly operations which are totally cosmetic. Nevertheless, exceptions do exist and convincing arguments may support medical-aesthetic measures with children and adolescents to avoid stigmatization in selected cases.[10]

Heightened attention to traditional duties and new attention to proposed responsibilities will enhance patient safety and choice.[11] It is not always possible to disregard the fact that what really motivates social changes in attitudes and demands for our intervention, is not for us to decide and there is no need to feel guilty either, when our technical gesture is aesthetic.[12]

Establishing an ethical code, which would entail all necessary moral sanctions in aesthetic surgery, would act favourably in the protection of those surgeons who perform their work according to the moral and ethical principles of their profession.[1]
